# Supplementary material for: Dermatophagoides farinae Extract Induces Interleukin 33-Mediated Atopic Skin Inflammation via Activation of RIP1
Source: Int J Mol Sci. 2023 Mar 9;24(6):5228. doi: 10.3390/ijms24065228 (PMC10049056; doi:10.3390/ijms24065228)
Supplement: Supplementary file 1 [file ijms-24-05228-s001.zip › ijms-2256920-supplementary.pdf]

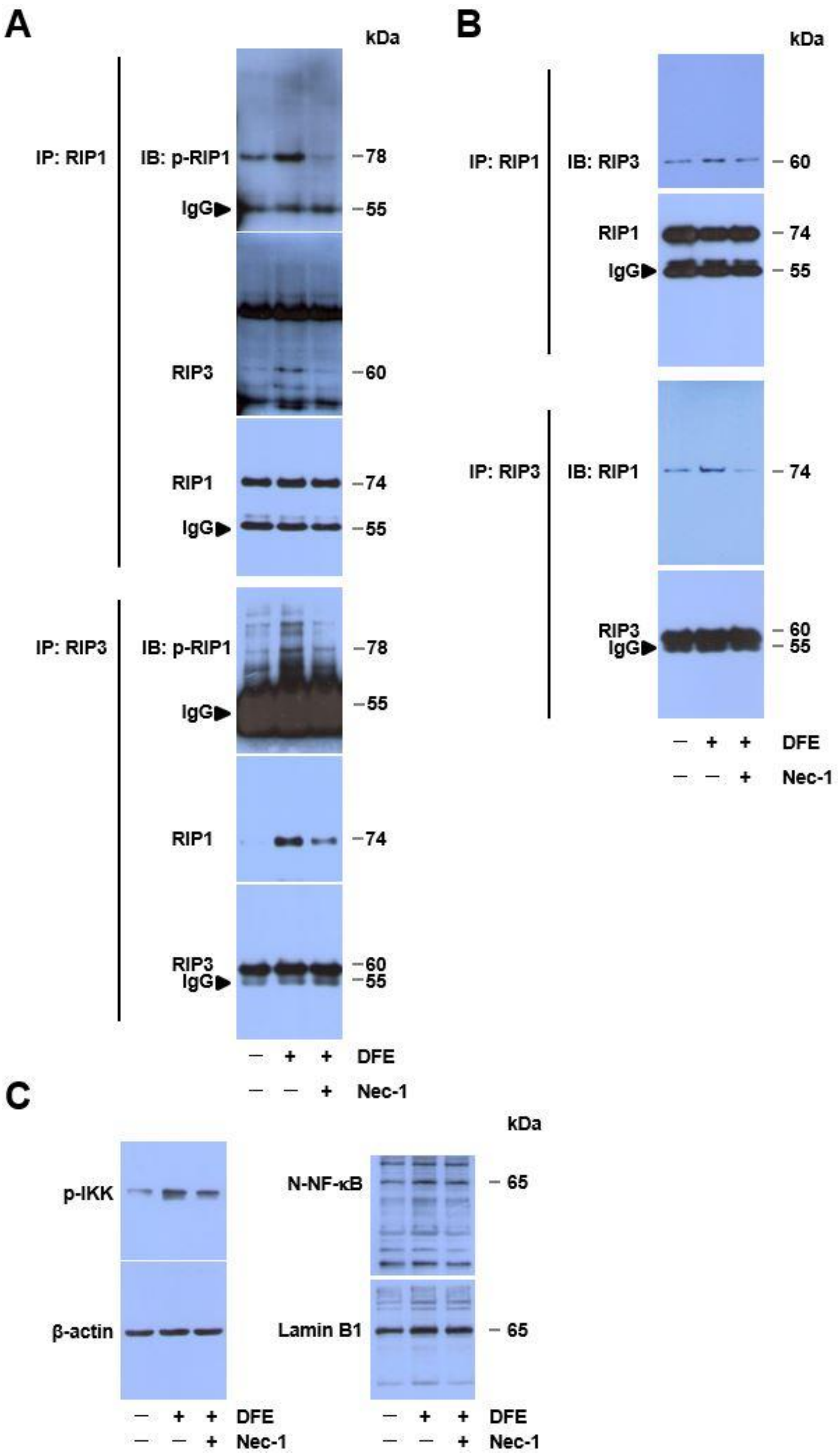

Supplementary Figure S1. (**A** and **B**) *HaCaT* cells or HKCs were stimulated with DFE (100 μg/ml) for immunoprecipitation (IP) with anti-phospho-RIP1, anti-RIP1, or anti-RIP3 antibodies. Phospho-RIP1, RIP1, and RIP3 protein levels from whole-cell lysates were determined using immunoblotting. (**C**) HKCs were pretreated with Nec-1 (10 μM) 1 h before stimulation with DFE (100 μg/ml) for 1 h. The activation of IKK and NF-κB were analyzed with immunoblotting. β-actin or lamin B1 were used as loading controls. N-NF-κB: nucleus NF-κB

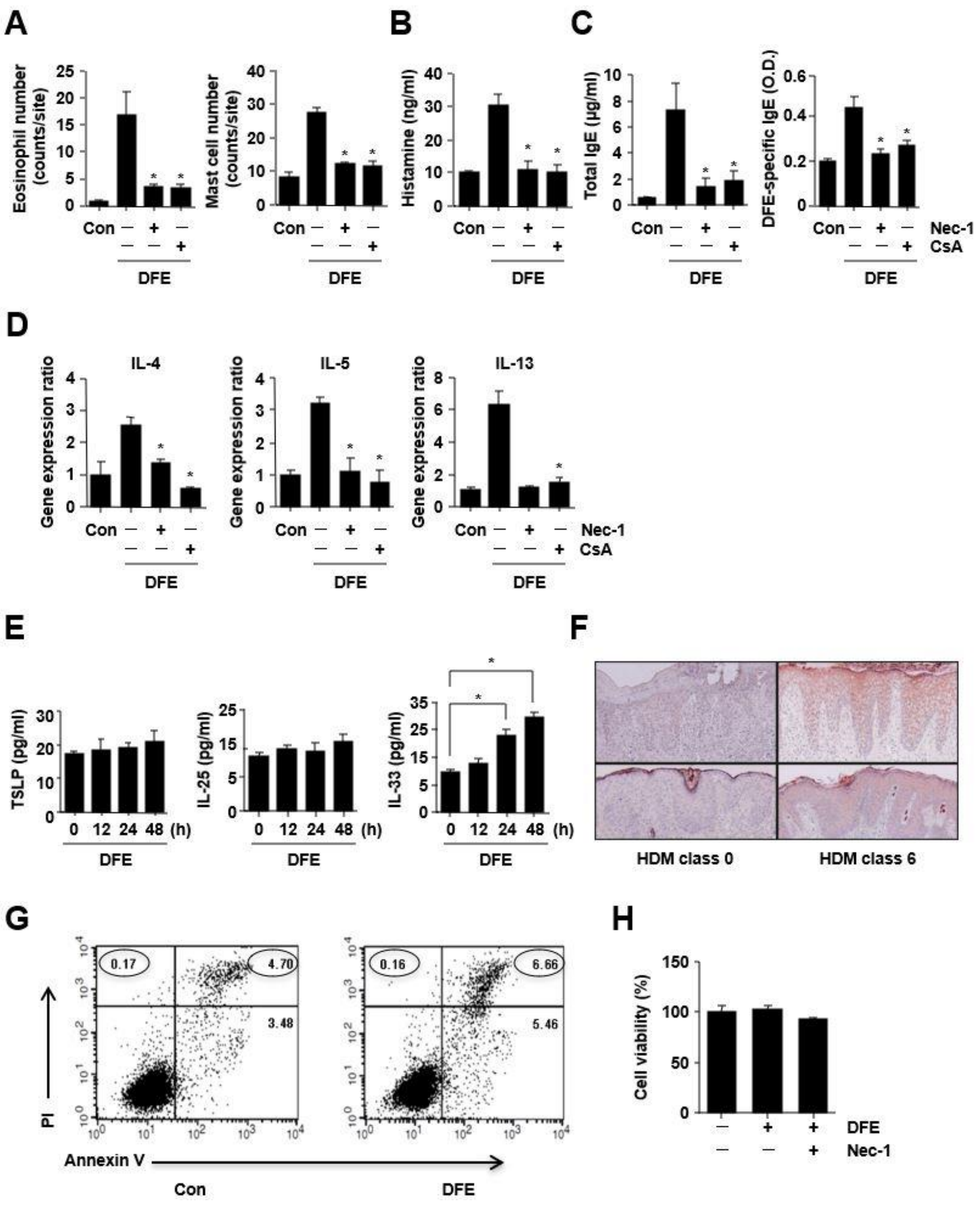

Supplementary Figure S2. (**A**) Eosinophils or mast cells were counted in 10 high-power fields at a magnification of ×400 in H&E or TE stained sections. (**B**) Histamine levels were detected using a fluorescent plate reader. (**C**) Serum levels of immunoglobulin in DFE-induced atopic skin inflammation. Total serum IgE levels and DFE-specific IgE levels were measured using ELISA. (**D**) Expression of Th2 pro-inflammatory cytokines in the ear of in the DFE-induced mouse model. Ears were excised, and total RNA was isolated. qPCR was performed as described in the Methods section. Samples of vehicle and DFE plus Nec-1 (5 mg/kg) or CsA (5 mg/kg) groups were collected on day 42. (**E**) *HaCaT* cells were stimulated with DFE (100 μg/ml) for 12 h, 24 h, or 48 h and the expression of IL-25 and IL-33 were measured using ELISA. (**F**) Tissue expression of IL-33 was detected by immunohistochemical staining in the lesional skin of AD patients with high house dust mite (HDM) sensitization (HDM-specific IgE levels > 50 IU/ml, class 5 & 6) and patients with no HDM sensitization (HDM-specific IgE levels < 0.7 IU/ml, class 0 & 1). (**G**) Flow cytometry analysis of HKCs treated with DFE (100 μg/ml) for 24 h. (h)

Cell viability of DFE and/or Nec-1 on HKCs. Cell viability was determined using the MTT assay. Data are presented as mean ± SEM (n = 5). *$p < 0.05$, significantly lower than the DFE group.

Supplementary Table S1. Clinical and laboratory characteristics of patients with AD for immunohistochemical analysis

	High HDM sensitization (*n* = 6)	Low HDM sensitization (*n* = 5)
Sex	Male (*n* = 5), female (*n* = 1)	Male (*n* = 2), female (*n* = 3)
Age*	19.8 ± 3.6	28.2 ± 14.3
AD Severity* (by EASI†)	25.9 ± 8.8	22.2 ± 8.2
Combined allergic diseases	Allergic rhinitis & asthma (*n* = 1)	None
Total IgE (kU/l)*	3013.0 ± 1871.0	816.8 ± 575.6
Total eosinophil count(/mm^3)*	532.0 ± 185.3	197.5 ± 124.3

AD, atopic dermatitis; HDM, house dust mite.

The HDM-specific IgE level was classified into seven quantitative classes by the following criteria: class 0, below 0.35 IU/ml; class 1, 0.35 to 0.69 IU/ml; class 2, 0.7 to 3.49 IU/ml; class 3, 3.5 to 17.49 IU/ml; class 4, 17.5 to 49.99 IU/ml; class 5, 50 to 99.99 IU/ml; and class 6, above 100 IU/ml. The patients were divided into two groups, the low sensitization group, composed of HDM-specific IgE classes 0&1, and the high sensitization group, composed of classes 5&6.

*Mean ± SD
†EASI, Eczema Area and Severity Index

Supplementary Table S2. List of primers for the qPCR experiment

	Gene	Forward primer (5′-3′)	Reverse primer (5′-3′)
Mouse	*Il-4*	ACA GGA GAA GGG ACG CCA T	GAA GCC GTA CAG ACG AGC TCA
	Il-5	GAA GTG TGG CGA GGA GAG AC	GCA CAG TTT TGT GGG GTT TT
	Il-13	GCA ACA TCA ACA GGA CCA GA	GTC AGG GAA TCC AGG GCT AC
	β-actin	TAG ACT TCG AGC AGG AGA TG	TTG ATC TTC ATG GTG CTA GG
